# Increasing procaspase 8 expression using repurposed drugs to induce HIV infected cell death in *ex vivo* patient cells

**DOI:** 10.1371/journal.pone.0179327

**Published:** 2017-06-19

**Authors:** Rahul Sampath, Nathan W. Cummins, Sekar Natesampillai, Gary D. Bren, Thomas D. Chung, Jason Baker, Keith Henry, Amélie Pagliuzza, Andrew D. Badley

**Affiliations:** 1 Division of Infectious Disease, Mayo Clinic Rochester, Rochester, MN, United States of America; 2 Office of Translation to Practice, Mayo Clinic Rochester, Rochester, MN, United States of America; 3 Division of Infectious Diseases, University of Minnesota, Minneapolis, MN, United States of America; 4 HIV Program, Hennepin County Medical Center, Minnneapolis, MN, United States of America; 5 Département de microbiologie, infectiologie et immunologie, Université de Montréal, Montréal, Canada; University Hospital Zurich, SWITZERLAND

## Abstract

HIV persists because a reservoir of latently infected CD4 T cells do not express viral proteins and are indistinguishable from uninfected cells. One approach to HIV cure suggests that reactivating HIV will activate cytotoxic pathways; yet when tested *in vivo*, reactivating cells do not die sufficiently to reduce cell-associated HIV DNA levels. We recently showed that following reactivation from latency, HIV infected cells generate the HIV specific cytotoxic protein Casp8p41 which is produced by HIV protease cleaving procaspase 8. However, cell death is prevented, possibly due to low procaspase 8 expression. Here, we tested whether increasing procaspase 8 levels in CD4 T cells will produce more Casp8p41 following HIV reactivation, causing more reactivated cells to die. Screening 1277 FDA approved drugs identified 168 that increased procaspase 8 expression by at least 1.7-fold. Of these 30 were tested for anti-HIV effects in an acute HIV_IIIb_ infection model, and 9 drugs at physiologic relevant levels significantly reduced cell-associated HIV DNA. Primary CD4 T cells from ART suppressed HIV patients were treated with one of these 9 drugs and reactivated with αCD3/αCD28. Four drugs significantly increased Casp8p41 levels following HIV reactivation, and decreased total cell associated HIV DNA levels (flurbiprofen: p = 0.014; doxycycline: p = 0.044; indomethacin: p = 0.025; bezafibrate: P = 0.018) without effecting the viability of uninfected cells. Thus procaspase 8 levels can be increased pharmacologically and, in the context of HIV reactivation, increase Casp8p41 causing death of reactivating cells and decreased HIV DNA levels. Future studies will be required to define the clinical utility of this or similar approaches.

## Introduction

There is no cure for HIV infection because no available therapy kills the reservoir of longlived, latently infected, predominantly central memory CD4 T cells [1, 2] that are responsible for viral rebound after combination antiretroviral therapy (cART) is discontinued. HIV persists in central memory CD4 T cells (TCM) that maintain a cell death resistant phenotype[3] which facilitates their persistence as historical archives of past immune responses. Indeed, our recent next-generation sequencing data in CD4 T cell subsets showed that, in contrast to effector memory CD4 T cells (TEM), TCM upregulates 6 proliferation genes, and down regulates 46 cell death genes by at least 2 fold change respectively[4]. Other groups have similarly observed lower levels of transcripts for 10 apoptosis regulatory genes including Caspase 8 and Caspase 3 in TCM from HIV-uninfected patients in comparison to TEM[5]. This reduction in proapoptotic molecule expression in concert with upregulation of anti-apoptotic molecules such as Bcl-xL or c-IAPs in TCM together prevents activation induced cell death (AICD)[6, 7]-one mechanism of homeostatic control of an activated immune response. Thus, it is likely that TCM resist apoptosis induced by a variety of death stimuli in comparison to TEM, including activation of the intrinsic pathway of cell death and death receptor ligation [8–10]. Indeed when TEM and TCM from the same donors are compared, TCM death in response to the Fas receptor ligation or in response to chemotherapy is less than in TEM, indicating a general death resistance[5, 11].

Death of HIV infected cells is induced by different pathways than uninfected (bystander) cells [12]. In uninfected CD4 T cells, aberrant immune activation, bacterial translocation across the gut epithelium and interaction with death inducing ligands (such as Fas Ligand or TNF Related Apoptosis Inducing Ligand (TRAIL)) or soluble HIV proteins (e.g., gp120, Tat, or Vpr) all contribute to cell loss [13–19]. In contrast, infected CD4 T cells can be killed by: RIG-I mediated sensing of HIV RNA[20], IFI-16 sensing of unintegrated HIV DNA [21] and DNA-PK-sensing of HIV integrase nicking of host DNA[22], all of which can induce apoptosis (or pyroptosis) of the infected cell. Once HIV is integrated into the host DNA, it can remain in a latent state for years, or may reactivate and produce progeny virions. With HIV replication, HIV protease in the cytosol cleaves both viral and host substrates[23–27], also leading to apoptosis. Since this pathway of HIV-infected cell killing occurs after integration in the HIV life cycle, it is important when HIV is reactivated from latently infected T cells. We have shown that HIV protease requires procaspase 8 to initiate apoptosis, and HIV protease cleavage of procaspase 8 generates Casp8p41; this, in turn, unmasks a latent BH3-like domain within Casp8p41 which binds to and activates Bak pore function causing increased mitochondrial outer membrane permeability (MOMP) and apoptosis [23, 28, 29]. In cells from HIV viremic patients, the presence of Casp8p41 predicts ongoing CD4 T cell loss [30], thereby attesting to the clinical relevance of this molecule.

When latently HIV infected CD4 T cells reactivate HIV, very few cells die despite producing progeny virions[31], suggesting that either Casp8p41 is not produced or that its apoptotic properties are antagonized. Our intensive studies of the molecular mechanisms by which Casp8p41 generated by HIV protease causes CD4 T cell death provides a conceptual framework by which we can test why TCM do not die despite HIV reactivation. We have previously shown that TCM from HIV-infected individuals in fact do generate Casp8p41 post reactivation, yet have a greater ratio of Bcl-2 to procaspase 8 than TEM, possibly explaining their resistance to HIV reactivation induced apoptosis [32]. Consistent with that model, we further showed that persistence of the latently infected cells can be reversed *ex vivo* by treating these cells with a Bcl-2 inhibitor, venetoclax [32]. Another potential way to enhance apoptosis of HIV infected CD4 T cells would be to increase generation of Casp8p41, which could be achieved by increasing procaspase 8 levels in the infected T cells. In the current report we test the hypothesis that increasing procaspase 8 increases the proportion of HIV infected cells that die after reactivation.

If a cure for HIV infection is to be widely applicable it must be simple, safe and scalable [33, 34]. A simple way to upregulate procaspase 8 expression in a CD4 T cell involves induction of immune activation[35]; but in the context of HIV infection, polyclonal immune activation may have negative untoward effects on HIV replication and immune function, including the induction of anergy. Instead, we have screened drugs already approved by the FDA for their ability to induced procaspase 8 expression in CD4 T cells and, if they do, whether treatment with these drugs results in an increased proportion of HIV infected cells dying after reactivation.

## Materials and methods

### Experimental design

The objective of this research was to determine if pharmacologic induction of procaspase 8 expression in latently HIV infected cells would increase cell death after viral reactivation and thereby decrease residual HIV DNA. The experimental design consisted of controlled laboratory experiments with the indicated number of replicates.

### Donors, reagents and CD4 cell isolation

Patient blood samples were approved through Mayo Clinic Institutional Review Board (IRB)-and Hennepin County Medical Center Human Subjects Committee approved protocols (#13-005646 and #1039-03), and written informed concent was obtained prior to study procedures. HIV positive patient samples were obtained either from leukapheresis or peripheral phlebotomy, whereas uninfected controls were obtained through apheresis leukocyte reduction cones[36]. All HIV positive donors were on combination antiretroviral therapy (cART) with HIV viral load <48 copies/mL. CD4 cells were isolated using RosetteSep™ Human CD4+ T Cell Enrichment Cocktail (negative selection) (Stemcell Technology Inc., Vancouver, CA) -per manufacture protocol. Media used for all experiments was RPMI1640 medium with L-glutamine (Gibco, Life Technologies) supplemented with 10% fetal bovine serum (FBS), penicillin (100 IU/mL) and streptomycin (100 mcg/mL).

### Drug screen to identify drugs that upregulate procaspase-8

CD4 T cells were isolated from HIV-uninfected donors by density gradient and negative selection as mentioned above. Cells are routinely >95% CD4 +. CD4 T cells (2x10^6^ cells /well) were cultured in 96-well plates pre-spotted with 50 nL of drug (10 mM stocks in neat DMSO) for 24 hours. Treated cells were harvested, lysed and cytoplasmic extracts assayed for procaspase8 by ELISA (Abcam) per manufacturer’s protocol. Fold change in procaspase 8 expression was determined by dividing procaspase 8 concentrations in lysates from individual drug treatments by the mean of untreated control wells. Drugs screened were from the Prestwich Chemical Library® (Illkirch-Graffenstaden, France) of 100% approved drugs (FDA, EMA and others).

### HIV infection, p24 measurement and reactivation

Primary CD4 T cells were infected with HIV-1_IIIb_ (NIH AIDS Reagent Program). Aliquots of the same pooled infectious supernatant were used for all experiments to ensure consistent MOIs across experiments. Primary CD4 cells were activated with IL-2 50 IU/mL and phytohaemagglutinin (PHA) 1 mcg/mL for 48hrs, then 100 x10^6^ cells were infected with 20 mL of viral stock for 6 hours with polybrene 10 mcg/mL. Infected cells were then washed twice and recultured in complete medium.

*Ex vivo* reactivation experiments were performed as follows. 2 to 5 million primary HIV patient bulk CD4 T cells were cultured in complete medium with or without procaspase-8 inducing drugs for 72 hours in the presence of tenofovir 10 micromolar and raltegravir 100 nM to prevent spreading infection. Cells were reactivated with plate bound αCD3 (clone OKT3) and soluble αCD28 (clone CD28.2) 1mcg/mL) for 48 hours prior to harvest. Drugs used to prime CD4T cells were used at concentrations that reflected serum peak concentrations in clinical studies as follows; carboplatin 39 mcg/mL[36], flurbiprofen 15.2 mcg/ml[37], doxycycline 10 mcg/mL[38], cinnarazine 200 ng/mL[39], indomethacin 2.4 mcg/mL[40], tenoxicam 2 mcg/mL[41], bezafibrate 14.3 mcg/mL[42], morantel 1.6 mcg/mL[43], retinoic acid 0.347 mcg/mL[44], acetaminophen 17.6 mcg/mL[45], triprolidine 0.015 mcg/mL[46], cholecalciferol 139 nM [47], ofloxacin 1.96 mcg/mL[48].

HIV P24 in the cell culture supernatant was measured by RETROTEK™ ELISA kits (Zeptometrix Corporation) according to manufacturer’s protocol.

Cell associated HIV-1 DNA was measured as follows—total DNA was extracted using the Qiagen DNeasy Blood and Tissue kit (Hilden, Germany) and analyzed by a real-time polymerase chain reaction (PCR) assay specific for HIV-LTR and β-globin primers. A standard curve of pNL4-3 plasmid from 10^6^ to 10 copies was used as an internal control. Briefly, 300 nM of the sense primer RU5-F 5′-TTAAGCCTCAATAAAGCTTGCC-3′ and the antisense primer RU5-R 5′- GTTCGGGCGCCACTGCTA GA -3′ were used in conjunction with 300 nM of the dual-labeled fluorogenic TaqMan probe 5′-FAM-CCAGAGTCACACAACAGACGG GCACA-TAMRA-3′. For a 20 microliter reaction, 10 microliters of gene expression master mix (Applied Biosystems) was used with 5 microliters of genomic DNA. PCR conditions consisted of one cycle of 95°C for 3 min followed by 45 cycles of 95°C for 15 s and 60°C for 1 min. Total HIV-1 DNA was compared and normalized with genomic DNA, determined by beta-globin detection using the Applied Biosystems (Carlsbad, CA). HIV-1 proviral DNA levels are expressed as HIV-1 copies/beta-globin genomic equivalent of 10^6^ cells.

### Procaspase 8 Western blots

Ten million cells were lysed in 100 microliter of lysis buffer (20 mM Tris-HCl [pH 7.5], 150 mM NaCl, 0.1% Triton X-100 [TX-100], 2 mcg/mL aprotinin, 10 mcg/mL leupeptin, 2 mcg/mL pepstatin, and1 mM phenylmethylsulfonyl fluoride [PMSF]) for 10 min on ice. For cell fractionation, the lysate was then further centrifuged at 15,000 × g for 5 min at 4°C, resulting in a pellet and cytosolic supernatant.

For Western blot analysis, 20 mcg of cell lysate was run on 10% polyacrylamide gels and then transferred onto polyvinylidene difluoride (PVDF) membranes for 2 h at 1,200 mA in transfer buffer (24 mM Tris, 192 mM glycine). The membranes were then blocked in Tris-buffered saline-Tween (TBST) (20 mM Tris, 150 mM NaCl, 0.05% Tween 20) with 2% bovine serum albumin (BSA) (Sigma, St. Louis, MO) overnight at 4°C. Membranes were blotted with the following primary antibodies: anti-actin (Sigma, St. Louis, MO), and monoclonal anti-caspase 8 (Biosource International, Camarillo, CA). Membranes were then washed three times with TBST, and a horseradish peroxidase-linked secondary antibody was used when necessary. All blots were developed by using a detection kit from Thermo Fisher Scientific (Waltham, MA).

### Flow cytometry

Flow cytometric measurement of intracellular Casp8p41 expression was performed as previously described using a neo-epitope specific antibody [32, 49]. Cell death was measured using LIVE/DEAD® fixable aqua dead cell stain (Invitrogen). Surface expression of CD25 and CD38 was determined by staining 1x10^6^ CD4 T cells with anti-CD25-PE (Becton Dickenson CAT 341010) and anti-CD38-PE (IMMUNOTECH CAT PNIM2371). Cells were washed and then resuspended in 200 microliters of PBS. Cells were then incubated at 4°C for 30min with the above antibodies, unstained control and isotype after which time they were washed and fixed with 2% paraformaldehyde. Proliferation was measured by staining with Ki67 per manufacturer’s protocol (BD Biosciences CAT 556026). FACS analysis was performed on either a FACScan or LSRII flow cytometer (BD Biosciences) based on multiparameter needs. FACS data was analyzed using FlowJo software (Tree Star Inc.).

### pGL4-Casp8 -luc reporter expression in primary CD4 T cells

For construction of caspase 8 promoter fragment (0.5 kB) luciferase-reporter plasmids we amplified using genomic DNA from jurkat cells as template by PCR approach and oligonucleotides that are flanked by KpnI and HindIII restriction sites. The resulting fragments were then cloned into the KpnI and HindIII sites of the pGL4-Basic vector (Promega). Promoter sequences amplified by PCR were confirmed by DNA sequencing using Mayo sequencing core lab. The TK-Renilla expression vector purchased from Promega (Madison, WI), was used as an internal control for transfections. Primary CD4 cells were transfected with 10 ug plasmid (9.5 mcg Casp8-luc and 0.5 mcg TK-renilla luc/10^6^ cells using an Electro Square Porator T820 (BTX, San Diego, CA) electroporator. Cells were then primed with Flurbiprofen, Indomethacin, Doxycycline, Bezafibrate, DMSO or PHA (positive control). Results are expressed as ratio of casp8-luc/Renilla expression at 24hours[50].

### Statistical analysis

Mean values of replicates were compared to control samples by unpaired t-tests, unless otherwise described. Figures depict mean (standard deviation) unless stated otherwise. An uncorrected alpha <0.1 was considered significant for advancement through drug screening procedures. For the final determination of select drug effect on HIV DNA, median values were compared using Kruskall-Wallis test with Dunn’s multiple comparison test, with corrected P values <0.05 considered statistically significant. Statistical analysis was performed using GraphPad Prism Version 6.

## Results

We sought to determine whether increasing procaspase 8 expression in CD4 T cells prior to reactivation would increase Casp8p41 production and death of HIV infected cells, resulting in reduced residual HIV cell associated DNA. To accomplish this, we adopted a three-phase screening approach. In the first phase, we screened a panel of FDA-approved drugs for the ability to increase procaspase 8 expression. In the second phase, we determined whether procaspase 8-inducing drugs decreased HIV DNA and/or replication in an acute, *in vitro* infection model using laboratory adapted HIV and primary CD4 T cells. Emphasis was placed on reductions in HIV DNA as opposed to viral replication as downstream assays and clinical applications would occur in the absence of active viral replication. In the final phase, we tested the effects of selected agents from the first two screens on *ex vivo* CD4 T cells from ART-suppressed, HIV positive patients, thereby determining the reproducibility of the results using multiple clinical isolates of HIV. At each screening step, drugs were advanced based on a reproducible biologic effect; plausible mechanism of action; anticipated drug safety, tolerability and clinical availability. Finally, mechanistic investigations confirmed upregulation of Casp8p41 expression associated with reduced HIV DNA after viral reactivation.

### Step 1: Screening for FDA approved drugs that upregulate procaspase 8 in primary CD4 T cells *in vitro*

**Fig 1:**
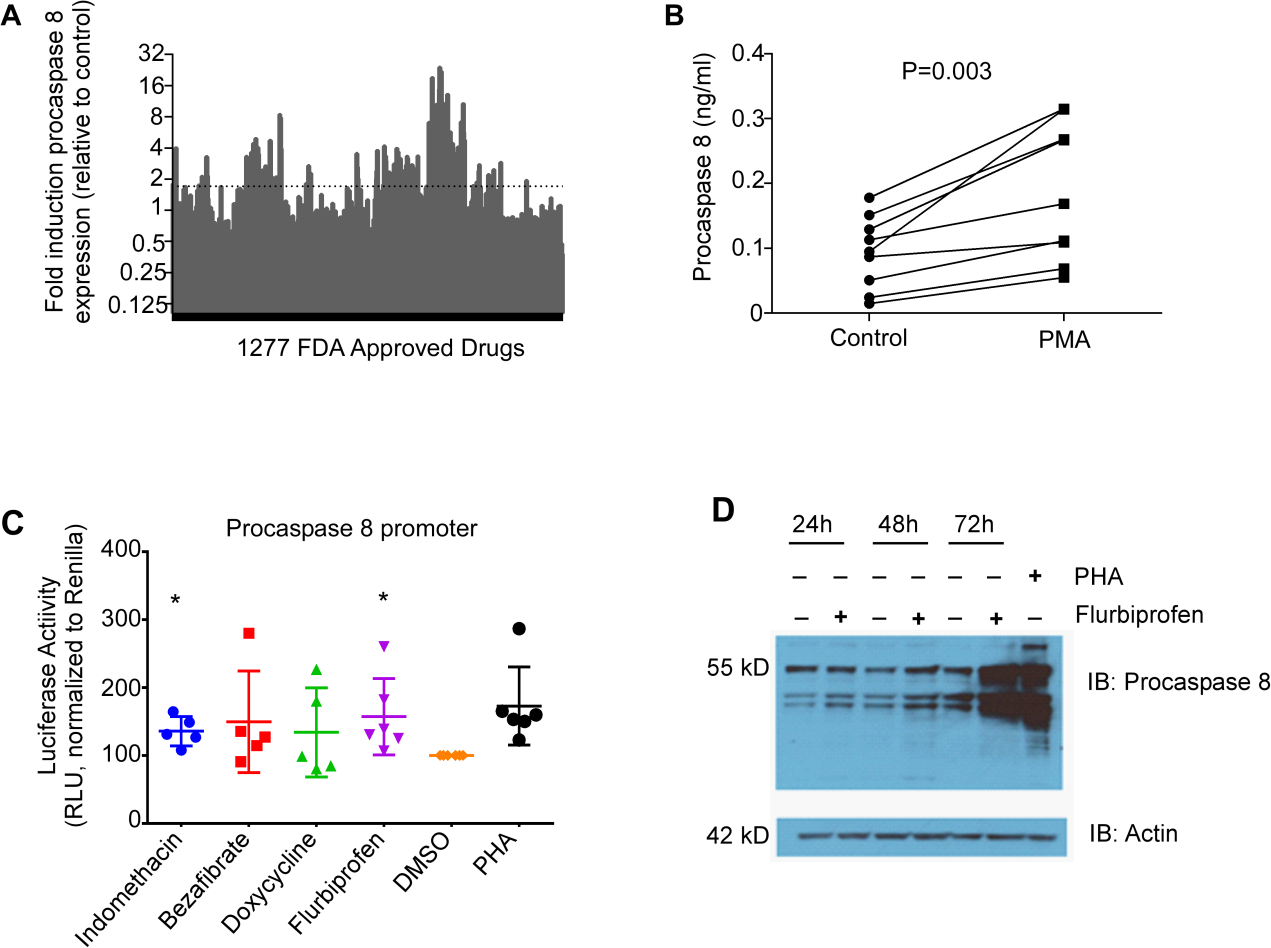
Select FDA approved drugs increase procaspase 8 expression. **A)** CD4 T cells were isolated from HIV-uninfected donors (9 total) and cultured in 96 well plates preloaded with 50 nL of drug (10mM in DMSO) for 24 hours. 1277 drugs from the Prestwick Chemical Library® collection of approved drugs were screened. Treated cells were assayed for procaspase8 by ELISA. **B)** PMA was used as a positive control for procaspase 8 induction. **C)** Primary uninfected CD4 cells from 5–6 different donors were transfected with pGL4-Casp8 -luc reporter and renilla then incubated for 24 hours with procaspase 8 inducing drugs. Luc/renilla ratios were measured and normalized to DMSO control. **D)** CD4 T cells from uninfected donors were primed with Flurbiprofen and cells were harvested at 24, 48 and 72 hours procaspase 8 measurement by western blot. PHA was used as a positive control.

According to Michalis–Menten enzyme kinetics [51], an increase in the procaspase 8 substrate concentration in the presence of catalytically active HIV protease will lead to a saturable increase in its Casp8p41 product concentration. A small molecule drug whose mechanism of action is upregulation of procaspase 8 expression would therefore lead to increased Casp8p41. However, we were unaware of any such drug and therefore, we screened 1277 FDA-approved drugs collection for the ability to increase procaspase 8 protein expression in primary CD4 T cells. CD4 T cells from HIV-uninfected donors were cultured in 96-well plates pre-spotted with 50 nL of drug (10 mM in DMSO) for 24 hours. Treated cells were harvested, lysed and cytoplasmic extracts assayed for procaspase 8 expression using a commercial ELISA and normalized to the mean of the untreated controls for the same plate (Fig 1A). Phorbol 12-myristate 13-acetate (PMA) was used as a positive control for increasing procaspase8 expression (Fig 1B)[52]. 168 (13%) drugs increased procaspase 8 expression in CD4 T cells by at least 1.7 fold, which represented the lower end of the 90% confidence interval of the geometric mean fold-induction in the PMA treated controls, and thus considered a biologically relevant induction. These drugs clustered in several therapeutic classes, including antimicrobials, non-steroidal anti-inflammatories (NSAID), histamine antagonists, and anti-neoplastic agents, among others. We next used an orthogonal (non-ELISA) and mechanistically independent (transcriptional reporter) secondary screen to validate the ELISA drug screen results. Primary CD4 T cells were transfected with a procaspase 8 promoter construct linked to luciferase, and these cells were treated with selected drugs that increased procaspase 8 by ELISA. Induction of procaspase 8 gene expression was then confirmed at the transcriptional level, in a subset of drugs identified in the ELISA screen to upregulate procaspase 8 protein expression, again using PHA as a positive control. Notably, both flurbiprofen and indomethacin (NSAIDs) significantly increased procaspase 8 gene expression compared to vehicle treated controls (p = 0.03 and 0.006, respectively), Fig 1C) consistent with previous reports with other NSAIDS [53–55]. In addition, resting primary CD4 T cells treated with flurbiprofen increased procaspase 8 protein expression compared to vehicle treated cells in a time dependent manner (Fig 1D), as assessed by western blotting. In summary, these results demonstrate that a significant number of FDA-approved drugs increase procaspase 8 gene and/or protein expression in CD4 T lymphocytes. Thirty of the 168 (17.8%, or 2.3% of the total collection) drugs were then selected for secondary screening for anti-HIV effects based on favorable side effect profiles and potentially plausible mechanisms of action.

### Step 2: Selected procaspase 8 inducing drugs decrease HIV DNA following acute primary CD4 T cell HIV infection *in vitro*

Casp8p41 is produced during acute HIV infection *in vitro* [56], and procaspase 8 expression is necessary for the death of HIV infected cells [23]. Furthermore, we have previously shown that induction of apoptosis in HIV infected cells reduces residual HIV virus and decreases HIV replication [57]. Therefore, we questioned whether increased procaspase 8 expression during acute HIV infection *in vitro* would alter and possibly decrease HIV replication and the number of cells containing cell associated HIV DNA.

**Fig 2:**
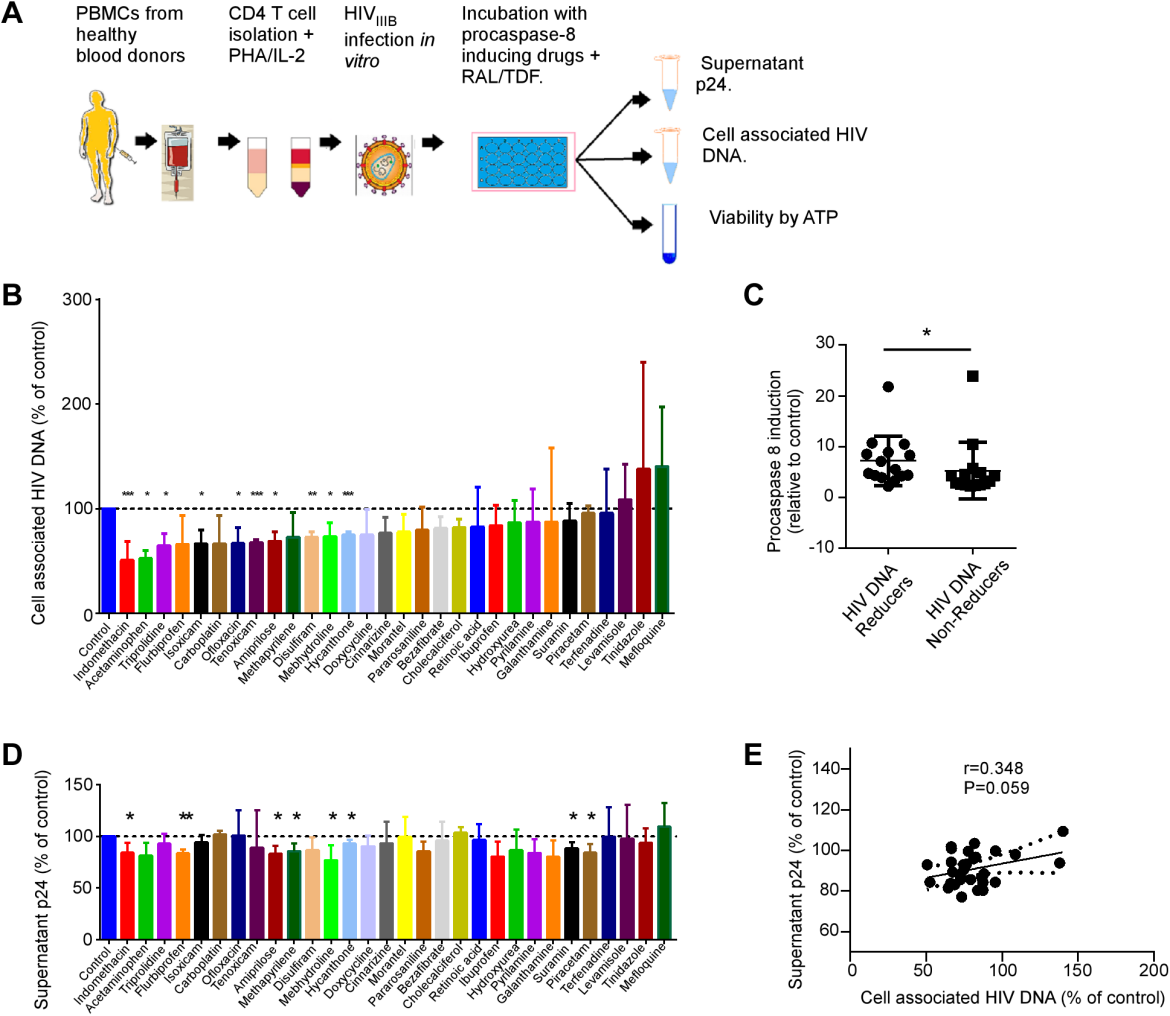
Procaspase 8 inducing drugs in acute *in vitro* HIV infection model. **A)** Primary uninfected CD4 cells from 3 donors were infected with HIV IIIB and then incubated with procaspase 8 inducing drugs for 48hours with RAL and TDF. ATP, HIV DNA by qPCR **(B)**, and supernatant P24 **(D)** were assessed. **C)** The degree of procaspase 8 expression induction by ELISA was compared between drugs which reduced HIV DNA or did not reduce HIV DNA in panel (B). Depicted are means (SD). E) Pearson correlation coefficient between the change in cell associated HIV DNA and change in supernatant p24 was assessed.

Activated primary CD4 T cells from 3 uninfected donors were infected with HIV IIIb *in vitro*, then cultured in the presence of one of the procaspase 8 inducing drugs identified above (or DMSO control), along with raltegravir (RAL) and tenofovir (TDF) to prevent spreading reinfection cycles (Fig 2A). Cell associated HIV DNA was measured by qRT-PCR (Fig 2B), and HIV p24 measured in cell culture supernatant by ELISA (Fig 2C) at 48 hours post infection. Nine of the procaspase 8 inducing drugs significantly decreased total HIV DNA levels compared to vehicle control treated cells: tiprolidine (-49%, P = 0.009), indomethacin (-48%, P = 0.0003), acetaminophen (-35%, P = 0.006), isoxicam (-34%, P = 0.01), ofloxacin(-33%, P = 0.02), tenoxicam(-32%, P = 0.0001), amiprilose (-31%, P = 0.03), disulfiram(-27%, P = 0.0009), mebhydroline(-27%, P = 0.02), hycanthone(-25%, P = 0.0001). In addition, the magnitude of change in HIV DNA in this model was associated with the degree of procaspase 8 induction. The fifteen drugs with the greatest reductions in HIV DNA had a higher procaspase 8 effect on the drug screen compared to the 15 drugs with the lowest reductions in HIV DNA (median [IQR] caspase 8 induction of 5.5 [4.2, 8.9] vs 3.2 [2.5, 4.9] fold respectively, P = 0.049 by Mann-Whitney test, Fig 2C).

Eight drugs also significantly decreased supernatant HIV p24 compared to vehicle control treated cells: indomethacin (-16%, P = 0.04), flurbiprofen (-16%, P = 0.001), amiprilose (-17%, P = 0.02), mebhydroline (-23%, P = 0.04), methapyriline (-14%, P = 0.03), hycanthone (-7%, P = 0.03), suramine (P = 0.02) and Piracetam (-12%, P = 0.02). This data suggests that some drugs which induce procaspase 8 expression reduce cell associated HIV DNA as well as HIV replication in an *in vitro* acute infection model, potentially through increasing apoptosis of HIV infected cells. In fact, there was a modest correlation between reductions in HIV DNA and supernatant p24 (r = 0.348, Fig 2E), although this did not reach statistical significance (P-0.059). Notably, total cell viability was not significantly reduced by any of the drugs tested. This is consistent with specificity of cell death to infected cells, as only a small percentage of all cells will contain HIV virus in this acute *in vitro* infection model [58].

### Step 3: Procaspase 8 inducing drugs which reduce HIV DNA following acute HIV infection reduce total HIV DNA following *ex vivo* HIV reactivation

**Fig 3:**
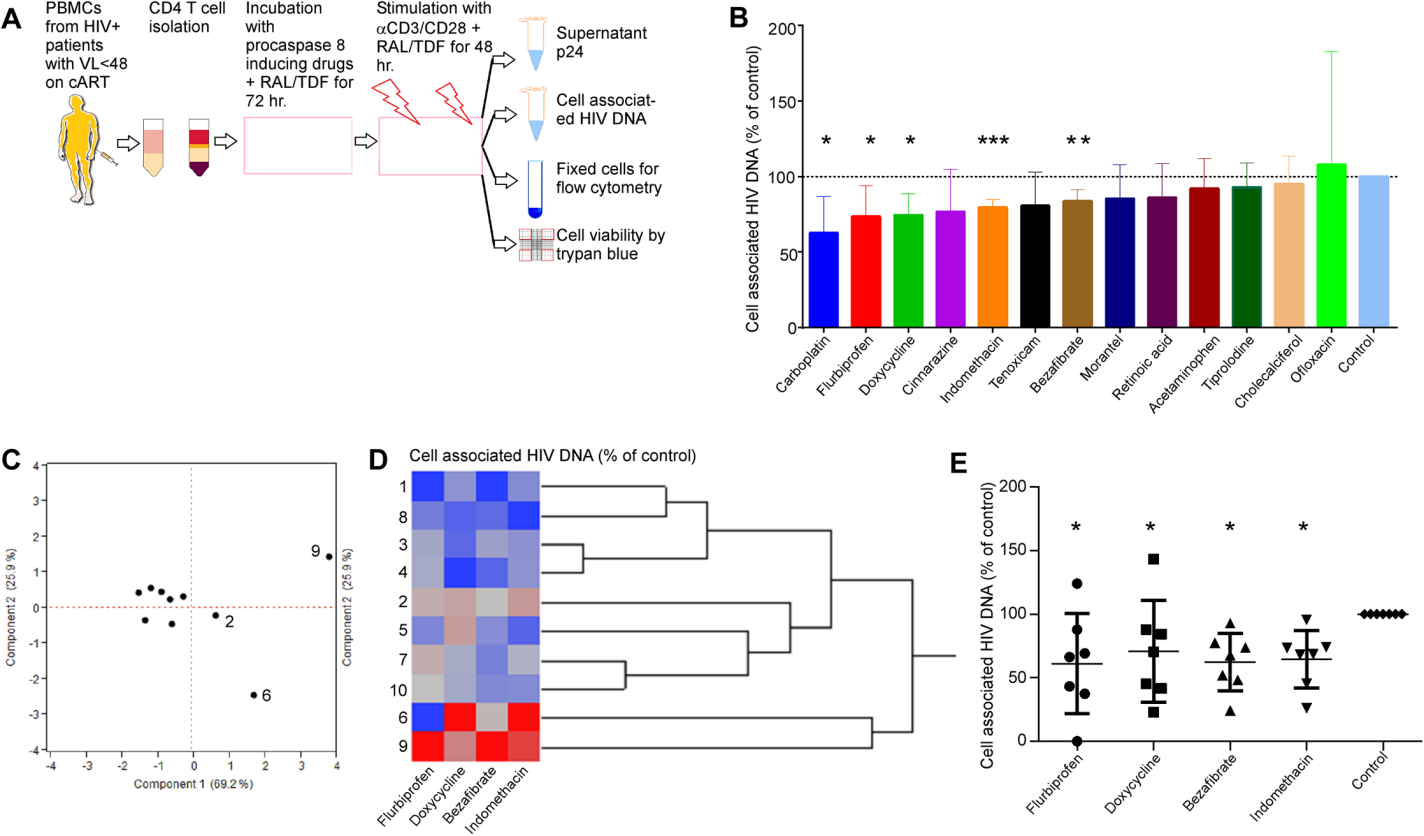
Procaspase 8 inducing drugs decrease total HIV DNA *ex vivo*. **A)** Primary CD4 cells from 5 HIV infected, virologically suppressed donors, on cART were obtained via leukapheresis and were primed for 72 hours with the indicated drugs, then reactivated with antiCD3/CD28. **B)** Cell associated HIV DNA was assessed by qPCR and normalized to DMSO treated control in the leukapheresis samples. Depicted are means (SD). **C/D/E)** 4 drugs—Flurbiprofen, Doxycycline, Indomethacin, and Bezafibrate—were selected to prime freshly obtained CD4 T cells from 10 HIV-infected patients for 72 h. These cells were then reactivated with antiCD3/CD28 and cell associated HIV DNA was assessed by qPCR. **C)** Principal component analysis of residual HIV DNA across the samples identified Patients 2, 6 and 9 as outliers. **D)** Hierarchical clustering of the same data confirmed Patients 2, 6 and 9 as outliers. **E)** Cell associated HIV DNA was compared between the indicated drugs and DMSO control for the remaining 7 treatment responders.

A treatment that reduces HIV DNA following HIV reactivation may be of use in contributing to a cure for HIV. We therefore used cells from cART suppressed HIV patients which will include a number of latently infected cells [59–64], and measured cell associated HIV DNA as a surrogate for HIV reservoir size, after treating these cells *ex vivo* with procaspase 8 inducing drugs followed by a reactivation stimulus. We assessed the effects of 13 drugs on HIV reservoir size using primary CD4 T cells (obtained by leukapheresis) from 5 HIV-infected patients who have had suppressed viral replication, following HIV reactivation. Bulk CD4 T cells, which comprise a mix of resting and activated cells at baseline, and T cell subsets, some of which would be latently infected, were isolated and cultured for 72 hours in the presence or absence of the above drugs, along with RAL/TFV to prevent spreading infection, then reactivated with antiCD3/CD28, and cell associated HIV DNA and supernatant HIV p24 levels were measured after 48h (Fig 3A). Five of the 13 drugs decreased cell associated HIV DNA significantly: Carboplatin (-37%; P = 0.008), Flurbiprofen (-27%; P = 0.01), Doxycycline (-26%; P = 0.01), Indomethacin (-23%; P = 0.0001), and Benzafibrate (-20%; P = 0.001) (Fig 3B). Supernatant HIV p24 was undetectable in all samples, consistent with the low frequency of infected cells in patients on suppressive ART, and the use of ART in the assay to prevent spreading infection. Due to potential toxicities of clinical use of carboplatin, this drug was not further evaluated.

*Ex vivo* CD4 T cells from an additional 10 freshly obtained HIV infected patient samples were treated with flurbiprofen, doxycycline, bezafibrate, indomethacin or vehicle control, reactivated as above, and total cell associated HIV DNA measured by qPCR after 48 hrs. We have previously shown interpatient variability in response to Casp8p41-targeting treatments in *ex vivo* cells [32]; specifically 8 of 11 patients’ cells treated with a Bcl-2 antagonist followed by reactivation responded with decreased HIV DNA. Anticipating similar variability in response to inducing procaspase 8 expression, we reasoned that mechanistically, if cells from an individual patient respond to procaspase 8 induction prior to reactivation by reducing HIV DNA, then similar effects should be seen across samples treated with the four drugs from distinct drug classes. Therefore, we first examined response to procaspase 8 induction for significant heterogeneity. Using a combination of principal component analysis [65, 66](Fig 3C) and unsupervised hierarchical clustering [67, 68](Fig 3D), patient samples 2, 6 and 9 were determined to be outliers in response to the procaspase-8 inducing agents, confirming our previous observations of variability in response to targeting Casp8p41-induced cell death. In the remaining 7 patient samples in which a consistent response was seen, cell associated HIV DNA was reduced significantly compared to control by flurbiprofen (adjusted p = 0.015), doxycycline (adjusted p = 0.045), bezafibrate (adjusted p = 0.019) and indomethacin (adjusted p = 0.026) (Fig 3E). Importantly, overall cell viability was not affected by treatment with procaspase-8 inducing drugs, consistent with selective death of a proportion of HIV-infected cells, which represent a small fraction of the total cell population. These data are consistent with a model wherein increasing procaspase 8 expression in all cells, yet generating Casp8p41 only in HIV infected cells which produce HIV protease, will result in the selective death of the HIV infected cells, leaving the non-HIV infected cells alive.

### Procaspase 8 activators which favor reduced HIV DNA following reactivation do not induce activation or proliferation

**Fig 4:**
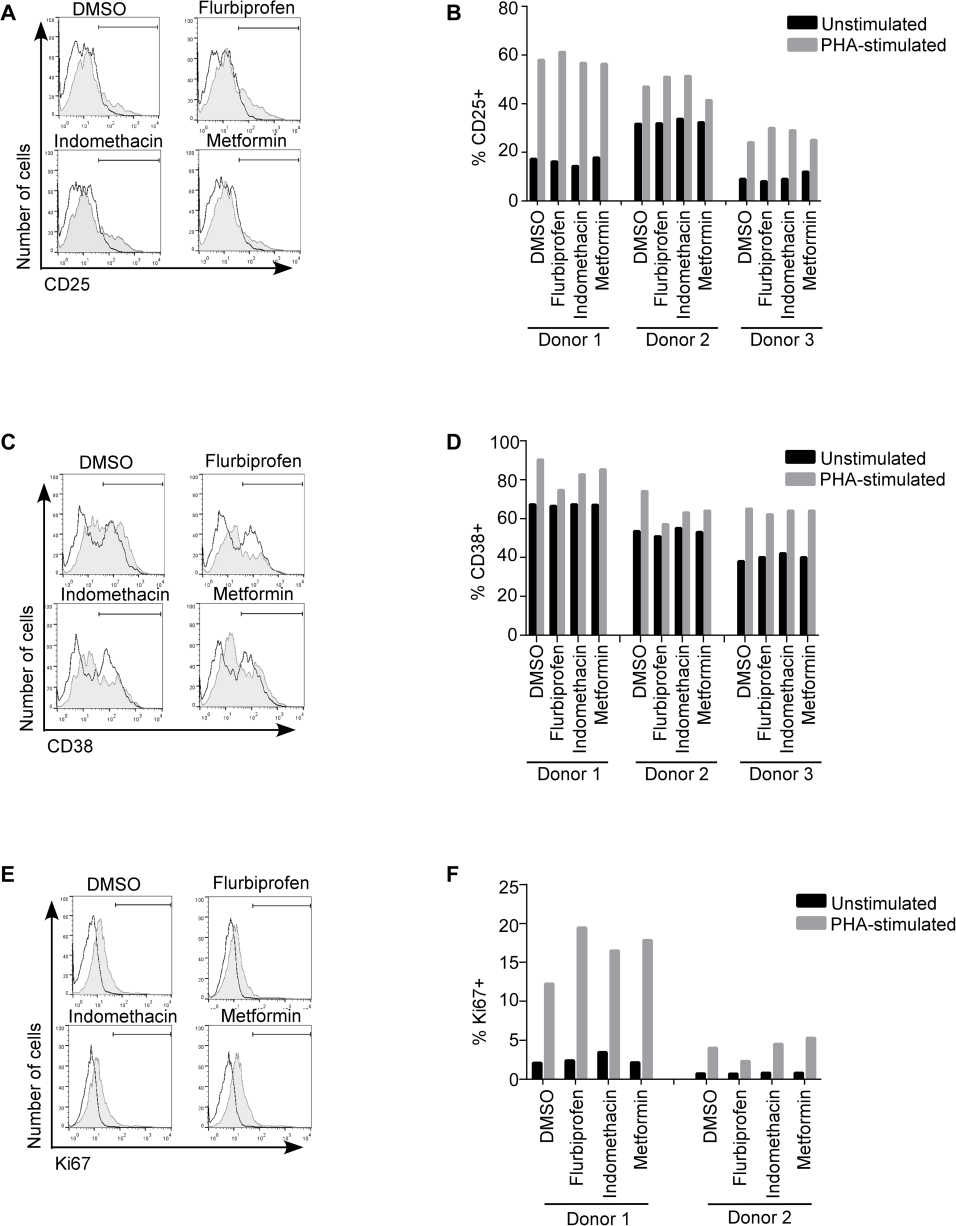
Procaspase 8 inducing drugs do not induce nonspecific activation. Primary uninfected CD4 cells were incubated with 2 drugs that induce procaspase 8 (flurbiprofen, and indomethacin), 1 drug that decreases procaspase 8 (metformin) or control (DMSO) with and without PHA stimulation. CD25 **(A, B)**, CD38 **(C, D)** and KI67 **(E, F)** expression levels were measured after incubation with the drugs for 48 h FACS. Black lines represent unstimulated samples, and shaded gray areas represent PHA-stimulated samples.

One proposed pathway inducing immune dysfunction in HIV disease is immune activation which negatively affects CD4 T cell homeostasis, effector immune mechanisms, viral replication and HIV associated disease complications [69–71]. Indeed, as was demonstrated in large clinical trials of IL2 therapy in HIV infected patients (ESPRIT, SILCAAT and STALWART trials), immune activation does not achieve the ultimate goals of viral control and clinically meaningful immune reconstitution [72–74]. Consequently, any therapies which are being considered for adjunctive treatment in HIV should neither induce excessive immune activation, nor impair physiologic activation with immune mediated viral clearance. Procaspase-8 is a critical signaling molecule in the induction of NF-kB dependent T cell activation via cell surface receptors [75]. Therefore, we assessed whether flurbiprofen and indomethacin, which increase procaspase-8 expression (Fig 1C) and cause reductions in HIV DNA following reactivation (Fig 3B and 3E) also effect immune activation. Primary uninfected CD4 T cells from 3 donors were treated with flurbiprofen, indomethacin, metformin (as a negative control since it decreased procaspase 8 in the drug screen) or control (DMSO) with or without stimulation with phytohemagglutinin (PHA). Cell activation was measured by surface staining for expression of CD25 and CD38 (3 donors), and proliferation assessed by intracellular expression on Ki67 stains (2 donors) after 48h. As expected, PHA treatment resulted in increased expression of CD25 (Fig 4A and 4B), CD38 (Fig 4C and 4D) and Ki67 (Fig 4E and 4F) compared to control cells. Treatment with flurbiprofen or indomethacin did not affect baseline, or change in response to PHA treatment, in CD25, CD38 or Ki67 expression in any subject. This *in vitro* data suggests that neither flurbiprofen nor indomethacin seem likely to abnormally modulate immune activation of CD4 T cells.

### Procaspase 8 activators which favor reduced HIV DNA following reactivation act by increasing Casp8p41 production following HIV reactivation

**Fig 5:**
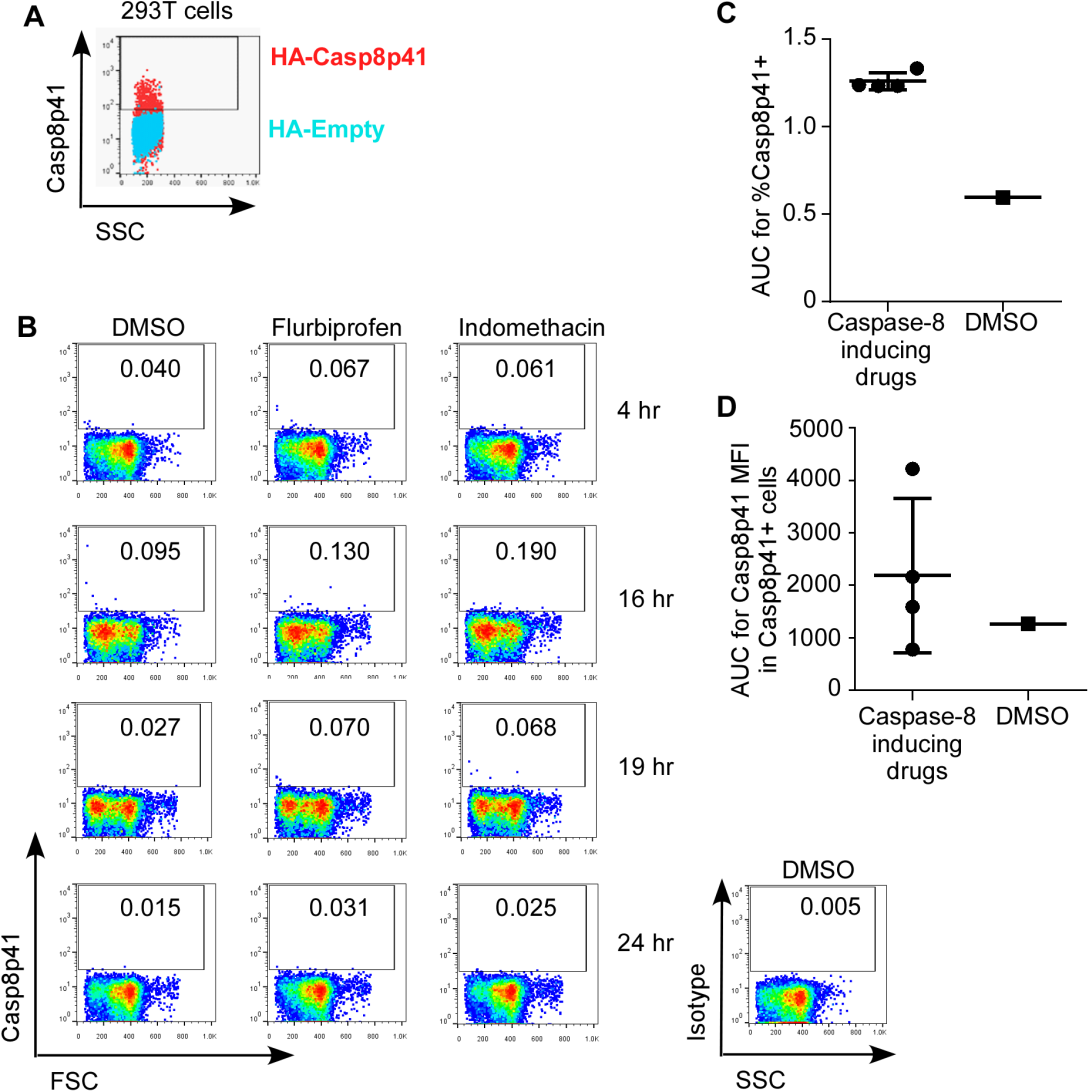
Procaspase 8 inducing drugs increase Casp8p41 expression *ex vivo*. **A) 293T cells transfected with HA-Casp8p41 (red) or HA-empty vector (blue) were stained with Casp8p41 mAb. Depicted is an overlay dot plot. B)** *Ex vivo* patient cells were primed with control and drugs for 72 hr, then reactivated with antiCD3/CD28, and harvested at time points thereafter (N = 2 per time point). These cells were then stained with Casp8p41 mAb and analyzed by FACS. **A)** Representative flow data for flurbiprofen and indomethacin treated samples at each time point. **B)** Area under the curve analysis for percent of CD4 T cells positive for Casp8p41 combining procaspase 8 inducing drugs versus vehicle treated controls. **C)** AUC analysis of MFI of Casp8p41 positive cells combining procaspase 8 inducing drugs versus vehicle treated controls.

The premise of our investigation was to induce procaspase 8 expression in CD4 T cells, positing that once that were achieved, when HIV is reactivated, Casp8p41 would be produced which would lead to increased apoptosis among the HIV reactivating cells (but not uninfected cells that do not contain HIV protease). Having identified treatments that can both induce procaspase 8 levels and decrease total cell associated HIV DNA levels post reactivation, we then assessed whether those treatments were associated with increased expression of Casp8p41 after reactivation. First, we confirmed the specificity of our neo-epitope specific antibody [56] against Casp8p41 (Fig 5A). Next, *ex vivo* HIV patient CD4 T cells were primed with vehicle control or caspase 8 inducing drugs, then reactivated with αCD3/αCD28 stimulation, and assessed for intracellular Casp8p41 expression using (N = 2 patients per time point). Casp8p41 expression, after HIV reactivation, was increased by caspase 8 inducing drugs compared to vehicle control, albeit to different degrees and at different time points between fluriprofen, indomethacin, bezafibrate and doxycycline (representative flow data Fig 5B). Casp8p41 expression was compared with caspase-8 inducing drugs and vehicle control two ways. First, the percentage of Casp8p41 positive cells over time by area under the curve analysis, showed a significant increase in Casp8p41expression with caspase-8 inducing drugs compared to vehicle control (Fig 5C, P = 0.0001). However, the second comparison, of the median fluorescence intensity (MFI) of Casp8p41+ cells after treatment with caspase-8 inducing, showed no difference in per-cell Casp8p41 expression level (Fig 5D, p = 0.29). This suggests that a minimal threshold of procaspase 8 expression is required for the generation of Casp8p41 by HIV protease activity, and that caspase-8 inducing drugs cause an increase in Casp8p41 expression after viral reactivation by raising the substrate level above that critical threshold.

## Discussion

HIV is currently effectively managed with cART, often with one pill daily, which results in suppressed viral replication, improved measures of immune function and improved life expectancy[76–79]. However it still remains desirable to develop a cure for HIV as even on cART, life expectancy for HIV infected patients is not completely normalized[80], comorbidities and accelerated rates of disease normally associated with ageing persist[81, 82], and the risk of transmission of HIV in the setting of detectable viral loads [82]. However given the global burden of HIV and its prevalence in resource limited settings, any curative treatment will need to be simple safe and scalable to be widely applicable [33]. Therefore, even if successful, the currently experimental treatments such as chimeric antigen receptor (CAR) T cells[83], CRISPR/Cas genome editing[84–87], or aggressive treatment of acute infection, are likely to have limited uptake in global clinical practice.

Drug repurposing involves studying drugs that are approved for one or more indications to see if they are safe and effective for other indications. A particular benefit of this approach is that much information exists on their pharmacology, toxicity and drug: drug interaction profile[88]. Moreover drug repurposing costs on average about $300 million and takes 6.5 years until FDA approval, as opposed to conventional drug development that takes twice the time and costs on average five times more[89]. A good example of this is with plerixafor, a drug initially developed as a CXCR4 antagonist to block HIV entry into CD4 T cells. During its development, treatment with this drug was noted to induce leukocytosis and increase the number of CD34 positive cells in the peripheral circulation. This drug has since been repurposed to mobilize stem cells prior to autologous stem cell transplants [90, 91].

For the past decade our group has studied HIV protease killing of infected CD4 T cells because very early in the HIV epidemic it was shown to be cytotoxic to both eukaryotic and prokaryotic cells–a fact that was exploited to screen for putative HIV protease inhibitors[26, 92–94]. Over that time, we discovered that HIV protease induced cytotoxicity is mediated by HIV protease cleavage of Procaspase 8 to generate a novel fragment Casp8p41[56]. Casp8p41 kills cells by virtue of the cleavage event unmasking a BH3 domain which binds a BH3 binding groove in Bak, to cause the activation of Bak, leading to loss of mitochondrial transmembrane potential and apoptosis[95]. Because Casp8p41 is formed by the action of HIV protease, Casp8p41 killing is specific to HIV infected, but not uninfected cells. Most recently we have discovered that Casp8p41 can bind other BH3 groove containing proteins such as Bcl-2, thereby providing a level of regulation of this death pathway[32]. Thus in cells with low Bcl-2, binding of Bak is favored resulting in cell death, whereas in cells with high Bcl-2, Casp8p41 binds Bcl-2, and cell death is averted.

Understanding this pathobiology of Casp8p41 allows testable hypotheses of how to modulate the pathway to favor death of HIV infected cells. For example the model presented suggests that HIV reactivation in the presence of a Bcl-2 antagonist will enhance the likelihood of HIV reactivating cells dying. Indeed, we observed that Bcl-2 inhibition with Venetoclax primes CD4 T cells to die upon HIV reactivation with consequent reductions in cell associated HIV DNA [32]. In this current report, we tested an alternate, but complementary hypothesis that increasing procaspase 8 levels above a critical threshold would increase the amount of Casp8p41 that is generated, resulting in death of the HIV reactivating cell. We observed that several drugs successfully increased procaspase 8 levels, and therefore we selected a subset of these drugs, which in our opinion might be used in HIV patients, based on their mechanisms of action, side effect profiles and pharmacology. That subset of drugs was further evaluated in models of HIV and we observed that anti-inflammatory agents and bezafibrate augment HIV infected cell death in primary *in vitro* infection, and reduced total cell associated HIV DNA in primary CD4 T cells from the majority, but not all, ART suppressed HIV patients sampled, following HIV reactivation, consistent with our previous data. It should be noted that additional agents were identified that increase procaspase 8 expression, and reduce HIV DNA in acute *in vitro* infection, such as amiprilose, mebhydroline, and hycathone (Fig 2), but were not further investigated in *ex vivo* patient cells. These additional agents may also deserve further investigation.

It is of interest to note that drugs from multiple classes and structures effect procaspase 8 levels. That these various drug types may impact HIV DNA levels may seem at first counterintuitive. However, in a large unbiased screen of over 500,000 drug combinations seeking anti HIV effects, multiple different Non-Steroidal anti-inflammatory drugs were identified to possess anti HIV properties [96], and in an unrelated study indomethacin decreased p24 production from HIV infected MT-2 cells [97]; our data provide a putative mechanism for these effects. Doxycycline is a tetracycline antibiotic that has anti-inflammatory properties[98], and in our hands also increases procaspase 8 levels.

As procaspase 8 is a central regulator of programmed cell death, basal and inducible expression of this important protein is subject to complex regulation. Mutational analyses of the Procaspase 8 promoter indicate that basal transcription is controlled by the transcription factor SP1, whereas inducible activity is controlled by the tumor suppressor P53[99]. NSAIDs have long been associated with tumor suppression and anticancer effects[100] including through activation of p53 dependant pathways [101]. Thus one potential mechanism by which NSAIDS might induce Procaspase 8 in T cells is through activation of p53 dependant procaspase 8 transcription. By contrast, Bezafibrate, via inhibition of proliferator-activated receptors (PPARα), was seen to significantly increase interferon-γ in patient with chronic hepatitis C after 4 months of therapy[102]. Because interferon gamma independently induces SP1 [103], Bezafibrate induced increases of procaspase 8 are likely to be mediated through SP1 upregulation acting upon the procaspase 8 promoter to increase basal transcription. Therefore, it is possible that the diverse procaspase 8-inducing drugs described herein exert their anti-HIV effect in diverse ways upstream of procaspase 8, but converging on a common final pathway of Casp8p41-induced, mitochondrial dependent apoptosis in infected cells.

Curing HIV will likely require a combinatorial approach that involves inhibiting HIV replication and homeostatic proliferation, boosting anti-HIV immune responses, reactivating HIV from latency, and promoting an apoptosis prone phenotype of the reactivating cell. It is unlikely that any one intervention could accomplish such a formidable task. Here, we have successfully identified an potential approach to the latter. We propose that further studies of repurposed drugs that increase procaspase 8 expression are warranted in other *in vitro* models and preclinical, *in vivo* models of HIV infection and latency that could be safely combined with other strategies to reduce HIV reservoir size. Further studies are also needed combining induced procaspase 8 expression and other latency reversal agents that may be more clinically relevant that T cell receptor stimulation.

## References

[1] Finzi D, Hermankova M, Pierson T, Carruth LM, Buck C, Chaisson RE, et al Identification of a Reservoir for HIV-1 in Patients on Highly Active Antiretroviral Therapy. Science. 1997;278(5341):1295–300. doi: 10.1126/science.278.5341.1295 9360927 10.1126/science.278.5341.1295

[2] Wong JK, Hezareh M, Günthard HF, Havlir DV, Ignacio CC, Spina CA, et al Recovery of Replication-Competent HIV Despite Prolonged Suppression of Plasma Viremia. Science. 1997;278(5341):1291–5. doi: 10.1126/science.278.5341.1291 9360926 10.1126/science.278.5341.1291

[3] Chomont N, El-Far M, Ancuta P, Trautmann L, Procopio FA, Yassine-Diab B, et al HIV reservoir size and persistence are driven by T cell survival and homeostatic proliferation. Nat Med. 2009;15(8):893–900. doi: 10.1038/nm.1972 19543283 10.1038/nm.1972 PMC2859814

[4] Cummins NW, Sainski AM, Dai H, Natesampillai S, Pang Y-P, Bren GD, et al Prime, shock, and kill: Priming CD4 T cells from HIV patients with a BCL-2 antagonist before HIV reactivation reduces HIV reservoir size. J Virol. 2016;90(8):4032–48. doi: 10.1128/JVI.03179-15 26842479 10.1128/JVI.03179-15 PMC4810548

[5] Riou C, Yassine-Diab B, Somogyi R, Greller LD, Gagnon D, Gimmig S, et al Convergence of TCR and cytokine signaling leads to FOXO3a phosphorylation and drives the survival of CD4+ central memory T cells. The Journal of experimental medicine. 2007;204(1):79–91. doi: 10.1084/jem.20061681 17190839 10.1084/jem.20061681 PMC2118424

[6] Xu L, Zhang L, Bertucci AM, Pope RM, Datta SK. Apigenin, a dietary flavonoid, sensitizes human T cells for activation-induced cell death by inhibiting PKB/Akt and NF-κB activation pathway. Immunology letters. 2008;121(1):74–83. doi: 10.1016/j.imlet.2008.08.004 18812189 10.1016/j.imlet.2008.08.004 PMC2610846

[7] Harari A, Vallelian F, Pantaleo G. Phenotypic heterogeneity of antigen‐specific CD4 T cells under different conditions of antigen persistence and antigen load. European journal of immunology. 2004;34(12):3525–33. doi: 10.1002/eji.200425324 15484193 10.1002/eji.200425324 PMC7163537

[8] Dunne PJ, Faint JM, Gudgeon NH, Fletcher JM, Plunkett FJ, Soares MVD, et al Epstein-Barr virus–specific CD8+ T cells that re-express CD45RA are apoptosis-resistant memory cells that retain replicative potential. Blood. 2002;100(3):933–40. doi: 10.1182/blood-2002-01-0160 12130505 10.1182/blood-2002-01-0160

[9] Grayson JM, Harrington LE, Lanier JG, Wherry EJ, Ahmed R. Differential sensitivity of naive and memory CD8+ T cells to apoptosis in vivo. The Journal of Immunology. 2002;169(7):3760–70. 12244170 10.4049/jimmunol.169.7.3760

[10] Gupta S, Bi R, Gollapudi S. Central Memory and Effector Memory Subsets of Human CD4+ and CD8+ T Cells Display Differential Sensitivity to TNF‐α‐Induced Apoptosis. Annals of the New York Academy of Sciences. 2005;1050(1):108–14. 16014525 10.1196/annals.1313.012

[11] Banz A, Pontoux C, Papiernik M. Modulation of Fas-dependent apoptosis: a dynamic process controlling both the persistence and death of CD4 regulatory T cells and effector T cells. The Journal of Immunology. 2002;169(2):750–7. 12097377 10.4049/jimmunol.169.2.750

[12] Cummins NW, Badley AD. Mechanisms of HIV-associated lymphocyte apoptosis: 2010. Cell death & disease. 2010;1(11):e99. 21368875 10.1038/cddis.2010.77 PMC3032328

[13] Hatano H, Jain V, Hunt PW, Lee T-H, Sinclair E, Do TD, et al Cell-based measures of viral persistence are associated with immune activation and programmed cell death protein 1 (PD-1)–expressing CD4+ T cells. Journal of Infectious Diseases. 2013;208(1):50–6. doi: 10.1093/infdis/jis630 23089590 10.1093/infdis/jis630 PMC3666131

[14] Marchetti G, Tincati C, Silvestri G. Microbial translocation in the pathogenesis of HIV infection and AIDS. Clinical microbiology reviews. 2013;26(1):2–18. doi: 10.1128/CMR.00050-12 23297256 10.1128/CMR.00050-12 PMC3553668

[15] Ipp H, Zemlin A. The paradox of the immune response in HIV infection: when inflammation becomes harmful. Clinica chimica acta. 2013;416:96–9. 10.1016/j.cca.2012.11.025 23228847

[16] Kotlyar D, Petrovas C, Cooper A, Ambrozak D, Annunziata C, Hernandez L, et al Potential Synergy of TRAIL with CDK9 Inhibition in Selective Killing of HIV Infected Cell Lines and Primary CD4+ T Cells. Blood. 2014;124(21):4142–.

[17] Kruman II, Nath A, Mattson MP. HIV-1 protein Tat induces apoptosis of hippocampal neurons by a mechanism involving caspase activation, calcium overload, and oxidative stress. Experimental neurology. 1998;154(2):276–88. doi: 10.1006/exnr.1998.6958 9878167 10.1006/exnr.1998.6958

[18] Haughey NJ, Mattson MP. Calcium dysregulation and neuronal apoptosis by the HIV-1 proteins Tat and gp120. Journal of acquired immune deficiency syndromes (1999). 2002;31:S55–61. 12394783 10.1097/00126334-200210012-00005

[19] Ferri KF, Jacotot E, Blanco J, Este JA, Kroemer G. Mitochondrial Control of Cell Death Induced by HIV‐1‐Encoded Proteins. Annals of the New York Academy of Sciences. 2000;926(1):149–64. 11193032 10.1111/j.1749-6632.2000.tb05609.x

[20] Solis M, Nakhaei P, Jalalirad M, Lacoste J, Douville R, Arguello M, et al RIG-I-mediated antiviral signaling is inhibited in HIV-1 infection by a protease-mediated sequestration of RIG-I. J Virol. 2011;85(3):1224–36. doi: 10.1128/JVI.01635-10 21084468 10.1128/JVI.01635-10 PMC3020501

[21] Doitsh G, Galloway NL, Geng X, Yang Z, Monroe KM, Zepeda O, et al Cell death by pyroptosis drives CD4 T-cell depletion in HIV-1 infection. Nature. 2014;505(7484):509–14. doi: 10.1038/nature12940 24356306 10.1038/nature12940 PMC4047036

[22] Cooper A, García M, Petrovas C, Yamamoto T, Koup RA, Nabel GJ. HIV-1 causes CD4 cell death through DNA-dependent protein kinase during viral integration. Nature. 2013;498(7454):376–9. doi: 10.1038/nature12274 23739328 10.1038/nature12274

[23] Nie Z, Bren GD, Rizza SA, Badley AD. HIV protease cleavage of procaspase 8 is necessary for death of HIV-infected cells. The open virology journal. 2008;2:1 doi: 10.2174/1874357900802010001 18818774 10.2174/1874357900802010001 PMC2548307

[24] Strack PR, Frey MW, Rizzo CJ, Cordova B, George HJ, Meade R, et al Apoptosis mediated by HIV protease is preceded by cleavage of Bcl-2. Proceedings of the National Academy of Sciences. 1996;93(18):9571–6. 10.1073/pnas.93.18.9571 PMC38469 8790371

[25] Kaplan A, Swanstrom R. The HIV-1 gag precursor is processed via two pathways: implications for cytotoxicity. Biomedica biochimica acta. 1990;50(4–6):647–53. 1801737

[26] ADAMS LD, TOMASSELLI AG, ROBBINS P, MOSS B, HEINRIKSON RL. HIV-1 protease cleaves actin during acute infection of human T-lymphocytes. AIDS research and human retroviruses. 1992;8(2):291–5. doi: 10.1089/aid.1992.8.291 1540415 10.1089/aid.1992.8.291

[27] Ventoso I, Blanco R, Perales C, Carrasco L. HIV-1 protease cleaves eukaryotic initiation factor 4G and inhibits cap-dependent translation. Proceedings of the National Academy of Sciences. 2001;98(23):12966–71. 10.1073/pnas.231343498 PMC60808 11606767

[28] Algeciras-Schimnich A, Belzacq-Casagrande A-S, Bren GD, Nie Z, Taylor JA, Rizza SA, et al Analysis of HIV protease killing through caspase 8 reveals a novel interaction between caspase 8 and mitochondria. The open virology journal. 2007;1:39 doi: 10.2174/1874357900701010039 18818773 10.2174/1874357900701010039 PMC2548302

[29] Sainski AM, Dai H, Natesampillai S, Pang Y-P, Bren GD, Cummins NW, et al Casp8p41 generated by HIV protease kills CD4 T cells through direct Bak activation. The Journal of cell biology. 2014;206(7):867–76. doi: 10.1083/jcb.201405051 25246614 10.1083/jcb.201405051 PMC4178959

[30] Cummins NW, Jiang W, McGinty J, Bren GD, Bosch RJ, Landay A, et al Intracellular Casp8p41 content is inversely associated with CD4 T cell count. Journal of Infectious Diseases. 2010;202(3):386–91. doi: 10.1086/653705 20565257 10.1086/653705 PMC2906377

[31] Shan L, Deng K, Shroff NS, Durand CM, Rabi SA, Yang HC, et al Stimulation of HIV-1-specific cytolytic T lymphocytes facilitates elimination of latent viral reservoir after virus reactivation. Immunity. 2012;36(3):491–501. Epub 2012/03/13. doi: 10.1016/j.immuni.2012.01.014 ; PubMed Central PMCID: PMC3501645. 22406268 10.1016/j.immuni.2012.01.014 PMC3501645

[32] Cummins NW, Sainski AM, Dai H, Natesampillai S, Pang Y-P, Bren GD, et al Prime, shock, and kill: Priming CD4 T cells from HIV patients with a BCL-2 antagonist before HIV reactivation reduces HIV reservoir size. J Virol. 2016:JVI. 03179–15. 10.1128/JVI.03179-15 PMC4810548 26842479

[33] Fauci AS, Marston HD, Folkers GK. An HIV cure: feasibility, discovery, and implementation. Jama. 2014;312(4):335–6. doi: 10.1001/jama.2014.4754 25038345 10.1001/jama.2014.4754

[34] Chun T-W, Moir S, Fauci AS. HIV reservoirs as obstacles and opportunities for an HIV cure. Nature immunology. 2015;16(6):584–9. doi: 10.1038/ni.3152 25990814 10.1038/ni.3152

[35] Dai C, Krantz SB. Interferon γ induces upregulation and activation of caspases 1, 3, and 8 to produce apoptosis in human erythroid progenitor cells. Blood. 1999;93(10):3309–16. 10233883

[36] Dietz AB, Bulur PA, Emery RL, Winters JL, Epps DE, Zubair AC, et al A novel source of viable peripheral blood mononuclear cells from leukoreduction system chambers. Transfusion. 2006;46(12):2083–9. doi: 10.1111/j.1537-2995.2006.01033.x 17176319 10.1111/j.1537-2995.2006.01033.x

[37] Davies NM. Clinical pharmacokinetics of flurbiprofen and its enantiomers. Clinical pharmacokinetics. 1995;28(2):100–14. doi: 10.2165/00003088-199528020-00002 7736686 10.2165/00003088-199528020-00002

[38] D Byron May P, BCPS Section Editor David C Hooper, MD Deputy Editor Elinor L Baron, MD, DTMH Tetracyclines. 2012.

[39] Nagarwal RC, Ridhurkar DN, Pandit J. In vitro release kinetics and bioavailability of gastroretentive cinnarizine hydrochloride tablet. AAPS PharmSciTech. 2010;11(1):294–303. doi: 10.1208/s12249-010-9380-5 20182827 10.1208/s12249-010-9380-5 PMC2850455

[40] monograph Cd. Tivorbex. Page current as of June 15th, 2016.

[41] Guentert T, Stebler T, Banken L, Defoin R, Schmitt M. Relative bioavailability of oral dosage forms of tenoxicam. Arzneimittel-Forschung. 1994;44(9):1051–4. 7986242

[42] Abshagen U, Bablok W, Koch K, Lang P, Schmidt H, Senn M, et al Disposition pharmacokinetics of bezafibrate in man. European journal of clinical pharmacology. 1979;16(1):31–8. 499297 10.1007/BF00644963

[43] Agency EM, Inspection VMa. MORANTEL: SUMMARY REPORT. 1998.

[44] Ozpolat B, Lopez-Berestein G, Adamson P, Fu C, Williams AH. Pharmacokinetics of intravenously administered liposomal all-trans-retinoic acid (ATRA) and orally administered ATRA in healthy volunteers. J Pharm Pharm Sci. 2003;6(2):292–301. 12935441

[45] summary Fe. McNeil's background package on acetaminophen for the September 19, 2002 Nonprescription Drugs Advisory Committee Meeting that was announced in the Federal Register of August 20, 2002.

[46] Miles MV, Balasubramanian R, Pittman AW, Grossman SH, Pappa KA, Smith MF, et al Pharmacokinetics of oral and transdermal triprolidine. The Journal of Clinical Pharmacology. 1990;30(6):572–5. 2355108 10.1002/j.1552-4604.1990.tb03623.x

[47] Petkovich M, Melnick J, White J, Tabash S, Strugnell S, Bishop CW. Modified-release oral calcifediol corrects vitamin D insufficiency with minimal CYP24A1 upregulation. The Journal of steroid biochemistry and molecular biology. 2015;148:283–9. doi: 10.1016/j.jsbmb.2014.11.022 25446887 10.1016/j.jsbmb.2014.11.022

[48] Adam D, Kees F. In vitro activity and concentrations in serum, urine, prostatic secretion and adenoma tissue of ofloxacin in urological patients. Drugs. 1987;34(1):44–50. 10.2165/00003495-198700341-00011 3481328

[49] Cummins NW, Neuhaus J, Sainski AM, Strausbauch MA, Wettstein PJ, Lewin SR, et al Short communication: CD4 T cell declines occurring during suppressive antiretroviral therapy reflect continued production of Casp8p41. AIDS research and human retroviruses. 2014;30(5):476–9. doi: 10.1089/AID.2013.0243 24344953 10.1089/aid.2013.0243 PMC4010171

[50] Bren GD, Trushin SA, Whitman J, Shepard B, Badley AD. HIV gp120 induces, NF-κB dependent, HIV replication that requires procaspase 8. PloS one. 2009;4(3):e4875 doi: 10.1371/journal.pone.0004875 19287489 10.1371/journal.pone.0004875 PMC2653723

[51] Michaelis L, Menten M. Die kinetik der invertinwirkung Biochem Z 49: 333–369. Find this article online. 1913.

[52] Hida A, Kawakami A, Nakashima T, Yamasaki S, Sakai H, Urayama S, et al Nuclear factor‐κB and caspases co‐operatively regulate the activation and apoptosis of human macrophages. Immunology. 2000;99(4):553–60. doi: 10.1046/j.1365-2567.2000.00985.x 10792503 10.1046/j.1365-2567.2000.00985.x PMC2327193

[53] Gómez-Lechón MJ, Ponsoda X, O’Connor E, Donato T, Castell JV, Jover R. Diclofenac induces apoptosis in hepatocytes by alteration of mitochondrial function and generation of ROS. Biochemical pharmacology. 2003;66(11):2155–67. 14609740 10.1016/j.bcp.2003.08.003

[54] Huang Y, He Q, Hillman MJ, Rong R, Sheikh MS. Sulindac sulfide-induced apoptosis involves death receptor 5 and the caspase 8-dependent pathway in human colon and prostate cancer cells. Cancer research. 2001;61(18):6918–24. 11559570

[55] Hossain MA, Kim DH, Jang JY, Kang YJ, Yoon J-H, Moon J-O, et al Aspirin induces apoptosis in vitro and inhibits tumor growth of human hepatocellular carcinoma cells in a nude mouse xenograft model. International journal of oncology. 2012;40(4):1298–304. doi: 10.3892/ijo.2011.1304 22179060 10.3892/ijo.2011.1304 PMC3584583

[56] Nie Z, Bren GD, Vlahakis SR, Schimnich AA, Brenchley JM, Trushin SA, et al Human immunodeficiency virus type 1 protease cleaves procaspase 8 in vivo. J Virol. 2007;81(13):6947–56. doi: 10.1128/JVI.02798-06 17442709 10.1128/JVI.02798-06 PMC1933285

[57] Shepard BD, De Forni D, McNamara DR, Foli A, Rizza SA, Abraham RS, et al Beneficial effect of TRAIL on HIV burden, without detectable immune consequences. PloS one. 2008;3(8):e3096 doi: 10.1371/journal.pone.0003096 18769477 10.1371/journal.pone.0003096 PMC2517653

[58] Jordan A, Bisgrove D, Verdin E. HIV reproducibly establishes a latent infection after acute infection of T cells in vitro. Embo J. 2003;22(8):1868–77. doi: 10.1093/emboj/cdg188 12682019 10.1093/emboj/cdg188 PMC154479

[59] Jain V, Hartogensis W, Bacchetti P, Hunt PW, Hatano H, Sinclair E, et al Antiretroviral therapy initiated within 6 months of HIV infection is associated with lower T-cell activation and smaller HIV reservoir size. Journal of Infectious Diseases. 2013;208(8):1202–11. doi: 10.1093/infdis/jit311 23852127 10.1093/infdis/jit311 PMC3778965

[60] Lee S, Chomont N, Fromentin R, Silicano R, Silicano J, Richman D, et al, editors. Anti-HIV antibody responses reflect the quantifiable HIV reservoir size. JOURNAL OF THE INTERNATIONAL AIDS SOCIETY; 2015: INT AIDS SOCIETY AVENUE DE FRANCE 23, GENEVA, 1202, SWITZERLAND.

[61] Buzon MJ, Martin-Gayo E, Pereyra F, Ouyang Z, Sun H, Li JZ, et al Long-term antiretroviral treatment initiated at primary HIV-1 infection affects the size, composition, and decay kinetics of the reservoir of HIV-1-infected CD4 T cells. J Virol. 2014;88(17):10056–65. doi: 10.1128/JVI.01046-14 24965451 10.1128/JVI.01046-14 PMC4136362

[62] Buzon MJ, Sun H, Li C, Shaw A, Seiss K, Ouyang Z, et al HIV-1 persistence in CD4+ T cells with stem cell-like properties. Nat Med. 2014;20(2):139–42. doi: 10.1038/nm.3445 24412925 10.1038/nm.3445 PMC3959167

[63] Siliciano JD, Siliciano RF. Recent developments in the effort to cure HIV infection: going beyond N = 1. Journal of Clinical Investigation. 2016;126(2):409 doi: 10.1172/JCI86047 26829622 10.1172/JCI86047 PMC4731192

[64] Halper-Stromberg A, Lu C-L, Klein F, Horwitz JA, Bournazos S, Nogueira L, et al Broadly neutralizing antibodies and viral inducers decrease rebound from HIV-1 latent reservoirs in humanized mice. Cell. 2014;158(5):989–99. doi: 10.1016/j.cell.2014.07.043 25131989 10.1016/j.cell.2014.07.043 PMC4163911

[65] Jackson DAaC, Y. Robust principal component analysis and outlier detection with ecological data. Environmetrics. 2004;15:129–39. doi: 10.1002/env.628

[66] Asa Ben-Hur IG. Detecting Stable Clusters Using Principal Component Analysis. Functional Genomics. 2003;224:159–82. 10.1385/1-59259-364-X:159 12710673

[67] Hodge VJaA J. A survey of outlier detection methodologies. Artificial Intelligence Review. 2004;22(2):85–126.

[68] Loureiro A, Torgo, L. and Soares, C. Outlier detection using clustering methods: a data cleaning application. Proceedings of KDNet Symposium on Knowledge-based Systems for the Public Sector Bonn, Germany. 2004.

[69] Fahey JL, Taylor JM, Manna B, Nishanian P, Aziz N, Giorgi JV, et al Prognostic significance of plasma markers of immune activation, HIV viral load and CD4 T‐cell measurements. AIDS. 1998;12(13):1581–90. 9764776 10.1097/00002030-199813000-00004

[70] Hazenberg MD, Otto SA, van Benthem BH, Roos MT, Coutinho RA, Lange JM, et al Persistent immune activation in HIV-1 infection is associated with progression to AIDS. AIDS. 2003;17(13):1881–8. doi: 10.1097/01.aids.0000076311.76477.6e 12960820 10.1097/00002030-200309050-00006

[71] Appay V, Sauce D. Immune activation and inflammation in HIV‐1 infection: causes and consequences. The Journal of pathology. 2008;214(2):231–41. doi: 10.1002/path.2276 18161758 10.1002/path.2276

[72] Markowitz N, Lopardo G, Wentworth D, Gey D, Babiker A, Fox L, et al Long-term effects of intermittent IL-2 in HIV infection: extended follow-up of the INSIGHT STALWART Study. PloS one. 2012;7(10):e47506 doi: 10.1371/journal.pone.0047506 23082173 10.1371/journal.pone.0047506 PMC3474747

[73] Emery S, Abrams DI, Cooper DA, Darbyshire JH, Lane HC, Lundgren JD, et al The Evaluation of Subcutaneous Proleukin®(interleukin-2) in a Randomized International Trial: rationale, design, and methods of ESPRIT. Controlled clinical trials. 2002;23(2):198–220. 11943448 10.1016/s0197-2456(01)00179-9

[74] Levy Y, editor Effect of interleukin-2 on clinical outcomes in patients with CD4+ cell count 50 to 299/mm3: primary results of the SILCAAT study. 16th Conference on Retroviruses and Opportunistic Infections; 2009.

[75] Su H, Bidère N, Zheng L, Cubre A, Sakai K, Dale J, et al Requirement for caspase-8 in NF-κB activation by antigen receptor. Science. 2005;307(5714):1465–8. doi: 10.1126/science.1104765 15746428 10.1126/science.1104765

[76] Bor J, Herbst AJ, Newell M-L, Bärnighausen T. Increases in adult life expectancy in rural South Africa: valuing the scale-up of HIV treatment. Science. 2013;339(6122):961–5. doi: 10.1126/science.1230413 23430655 10.1126/science.1230413 PMC3860268

[77] Samji H, Cescon A, Hogg RS, Modur SP, Althoff KN, Buchacz K, et al Closing the gap: increases in life expectancy among treated HIV-positive individuals in the United States and Canada. PloS one. 2013;8(12):e81355 doi: 10.1371/journal.pone.0081355 24367482 10.1371/journal.pone.0081355 PMC3867319

[78] Nakagawa F, May M, Phillips A. Life expectancy living with HIV: recent estimates and future implications. Current opinion in infectious diseases. 2013;26(1):17–25. doi: 10.1097/QCO.0b013e32835ba6b1 23221765 10.1097/QCO.0b013e32835ba6b1

[79] Romley JA, Juday T, Solomon MD, Seekins D, Brookmeyer R, Goldman DP. Early HIV treatment led to life expectancy gains valued at $80 billion for people infected in 1996–2009. Health Affairs. 2014;33(3):370–7. doi: 10.1377/hlthaff.2013.0623 24590933 10.1377/hlthaff.2013.0623

[80] Sabin CA. Do people with HIV infection have a normal life expectancy in the era of combination antiretroviral therapy? BMC medicine. 2013;11(1):1. 24283830 10.1186/1741-7015-11-251 PMC4220799

[81] Pathai S, Lawn SD, Gilbert CE, McGuinness D, McGlynn L, Weiss HA, et al Accelerated biological ageing in HIV-infected individuals in South Africa: a case–control study. Aids. 2013;27(15):2375–84. doi: 10.1097/QAD.0b013e328363bf7f 23751258 10.1097/QAD.0b013e328363bf7f PMC3805356

[82] High KP, Brennan-Ing M, Clifford DB, Cohen MH, Currier J, Deeks SG, et al HIV and aging: state of knowledge and areas of critical need for research. A report to the NIH Office of AIDS Research by the HIV and Aging Working Group. Journal of acquired immune deficiency syndromes (1999). 2012;60(Suppl 1):S1–18. 10.1097/QAI.0b013e31825a3668 PMC3413877 22688010

[83] Scholler J, Brady TL, Binder-Scholl G, Hwang W-T, Plesa G, Hege KM, et al Decade-long safety and function of retroviral-modified chimeric antigen receptor T cells. Science translational medicine. 2012;4(132):132ra53–ra53. doi: 10.1126/scitranslmed.3003761 22553251 10.1126/scitranslmed.3003761 PMC4368443

[84] Li L, Krymskaya L, Wang J, Henley J, Rao A, Cao L-F, et al Genomic editing of the HIV-1 coreceptor CCR5 in adult hematopoietic stem and progenitor cells using zinc finger nucleases. Molecular Therapy. 2013;21(6):1259–69. doi: 10.1038/mt.2013.65 23587921 10.1038/mt.2013.65 PMC3677314

[85] Ebina H, Misawa N, Kanemura Y, Koyanagi Y. Harnessing the CRISPR/Cas9 system to disrupt latent HIV-1 provirus. Scientific reports. 2013;3. 10.1038/srep02510 PMC3752613 23974631

[86] Hu W, Kaminski R, Yang F, Zhang Y, Cosentino L, Li F, et al RNA-directed gene editing specifically eradicates latent and prevents new HIV-1 infection. Proceedings of the National Academy of Sciences. 2014;111(31):11461–6. 10.1073/pnas.1405186111 PMC4128125 25049410

[87] Liao H-K, Gu Y, Diaz A, Marlett J, Takahashi Y, Li M, et al Use of the CRISPR/Cas9 system as an intracellular defense against HIV-1 infection in human cells. Nature communications. 2015;6. 10.1038/ncomms7413 25752527

[88] Strittmatter SM. Overcoming drug development bottlenecks with repurposing: old drugs learn new tricks. Nat Med. 2014;20(6):590–1. doi: 10.1038/nm.3595 24901567 10.1038/nm.3595 PMC4371131

[89] Nosengo N. Can you teach old drugs new tricks? Nature. 2016;534(7607):314 doi: 10.1038/534314a 27306171 10.1038/534314a

[90] Flomenberg N, Devine SM, DiPersio JF, Liesveld JL, McCarty JM, Rowley SD, et al The use of AMD3100 plus G-CSF for autologous hematopoietic progenitor cell mobilization is superior to G-CSF alone. Blood. 2005;106(5):1867–74. doi: 10.1182/blood-2005-02-0468 15890685 10.1182/blood-2005-02-0468

[91] DiPersio JF, Stadtmauer EA, Nademanee A, Micallef IN, Stiff PJ, Kaufman JL, et al Plerixafor and G-CSF versus placebo and G-CSF to mobilize hematopoietic stem cells for autologous stem cell transplantation in patients with multiple myeloma. Blood. 2009;113(23):5720–6. doi: 10.1182/blood-2008-08-174946 19363221 10.1182/blood-2008-08-174946

[92] Büttner J, Dornmair K, Schramm HJ. Screening of Inhibitors of HIV-1 Protease Using anEscherichia coliCell Assay. Biochemical and biophysical research communications. 1997;233(1):36–8. doi: 10.1006/bbrc.1997.6403 9144391 10.1006/bbrc.1997.6403

[93] Konvalinka J, Litterst MA, Welker R, Kottler H, Rippmann F, Heuser A-M, et al An active-site mutation in the human immunodeficiency virus type 1 proteinase (PR) causes reduced PR activity and loss of PR-mediated cytotoxicity without apparent effect on virus maturation and infectivity. J Virol. 1995;69(11):7180–6. 7474139 10.1128/jvi.69.11.7180-7186.1995 PMC189639

[94] Badley AD, Pilon AA, Landay A, Lynch DH. Mechanisms of HIV-associated lymphocyte apoptosis. Blood. 2000;96(9):2951–64. 11049971

[95] Sainski AM, Natesampillai S, Cummins NW, Bren GD, Taylor J, Saenz DT, et al The HIV-1-specific protein Casp8p41 induces death of infected cells through Bax/Bak. J Virol. 2011;85(16):7965–75. doi: 10.1128/JVI.02515-10 21653671 10.1128/JVI.02515-10 PMC3147983

[96] Tan X, Hu L, Luquette LJ III, Gao G, Liu Y, Qu H, et al Systematic identification of synergistic drug pairs targeting HIV. Nature biotechnology. 2012;30(11):1125–30. doi: 10.1038/nbt.2391 23064238 10.1038/nbt.2391 PMC3494743

[97] Bourinbaiar AS, Lee-Huang S. The non‐steroidal anti‐inflammatory drug, indomethacin, as an inhibitor of HIV replication. FEBS letters. 1995;360(1):85–8. 7875307 10.1016/0014-5793(95)00057-g

[98] Webster G, Del Rosso JQ. Anti-inflammatory activity of tetracyclines. Dermatologic clinics. 2007;25(2):133–5. doi: 10.1016/j.det.2007.01.012 17430750 10.1016/j.det.2007.01.012

[99] Liedtke C, Gröger N, Manns MP, Trautwein C. The human caspase-8 promoter sustains basal activity through SP1 and ETS-like transcription factors and can be up-regulated by a p53-dependent mechanism. Journal of Biological Chemistry. 2003;278(30):27593–604. doi: 10.1074/jbc.M304077200 12748179 10.1074/jbc.M304077200

[100] Rüegg C, Zaric J, Stupp R. Non steroidal anti‐inflammatory drugs and COX‐2 inhibitors as anti‐cancer therapeutics: hypes, hopes and reality. Annals of medicine. 2003;35(7):476–87. 14649330 10.1080/07853890310017053

[101] Zhang Y-J, Dai Q, Wu S-M, Zhu H-Y, Shen G-F, Li E-L, et al Susceptibility for NSAIDs-induced apoptosis correlates to p53 gene status in gastric cancer cells. Cancer investigation. 2008;26(9):868–77. doi: 10.1080/07357900801944872 18798056 10.1080/07357900801944872

[102] Knop V, Bergk A, Schlosser B, Thieringer J, van Bömmel F, Frost N, et al Bezafibrate maintenance therapy in patients with advanced chronic hepatitis C. European journal of gastroenterology & hepatology. 2013;25(5):594–600. 23325275 10.1097/MEG.0b013e32835cc878

[103] Harris SM, Harvey EJ, Hughes TR, Ramji DP. The interferon-γ-mediated inhibition of lipoprotein lipase gene transcription in macrophages involves casein kinase 2-and phosphoinositide-3-kinase-mediated regulation of transcription factors Sp1 and Sp3. Cellular signalling. 2008;20(12):2296–301. doi: 10.1016/j.cellsig.2008.08.016 18793716 10.1016/j.cellsig.2008.08.016 PMC2586094

